# Regulatory Mechanisms and Therapeutic Implications of Lysosomal Dysfunction in Alzheimer's Disease

**DOI:** 10.7150/ijbs.103028

**Published:** 2025-01-13

**Authors:** Yeji Kim, Tae-Young Ha, Myung-Shik Lee, Keun-A Chang

**Affiliations:** 1 Department of Health Sciences and Technology, Gachon Advanced Institute for Health Sciences & Technology, Gachon University, Incheon 21999, Korea.; 2 Department of Pharmacology, College of Medicine, Gachon University, Incheon 21999, Korea.; 3 Neuroscience Research Institute, Gachon University, Incheon 21565, Korea.; 4 Soonchunhyang Institute of Medi-bio Science & Division of Endocrinology, Department of Internal Medicine & Immunology, Soonchunhyang University College of Medicine, Cheonan 31151, Korea.; 5 Chief Scientific Officer, LysoTech, Inc., Seoul 03766, Korea.

**Keywords:** Alzheimer's disease, Autophagy-lysosomal pathway, Lysosomal dysfunction, Lysosomal stress response

## Abstract

Alzheimer's disease (AD) is characterized by the accumulation of amyloid-beta (Aβ) plaques, neurofibrillary tangles (NFTs) formed from hyperphosphorylated Tau, and widespread neuronal loss. The autophagy-lysosomal pathway plays a crucial role in maintaining cellular homeostasis by degrading and recycling of damaged organelles and aggregate amyloid proteins implicated in AD. Lysosomes are key effectors of autophagic process, responsible for the breakdown of a variety of damaged organelles and aggregate or dysfunctional proteins. This review examines the role of lysosomal dysfunction in AD pathophysiology, focusing on genetic factors, acidification abnormalities, and other contributing factors. We also explore the involvement of lysosomal dysfunction of microglia in AD pathology, and cover the role of lysosomal stress response (LSR) in cellular response to neuronal injury associated with AD. Furthermore, we discuss potential therapeutic strategies targeting lysosomal proteolysis pathway and addressing lysosomal dysfunction for AD treatment, including the pharmacologically activating lysosomal activity, regulating TFEB, and considering other emerging approaches.

## Introduction

**Figure 1:**
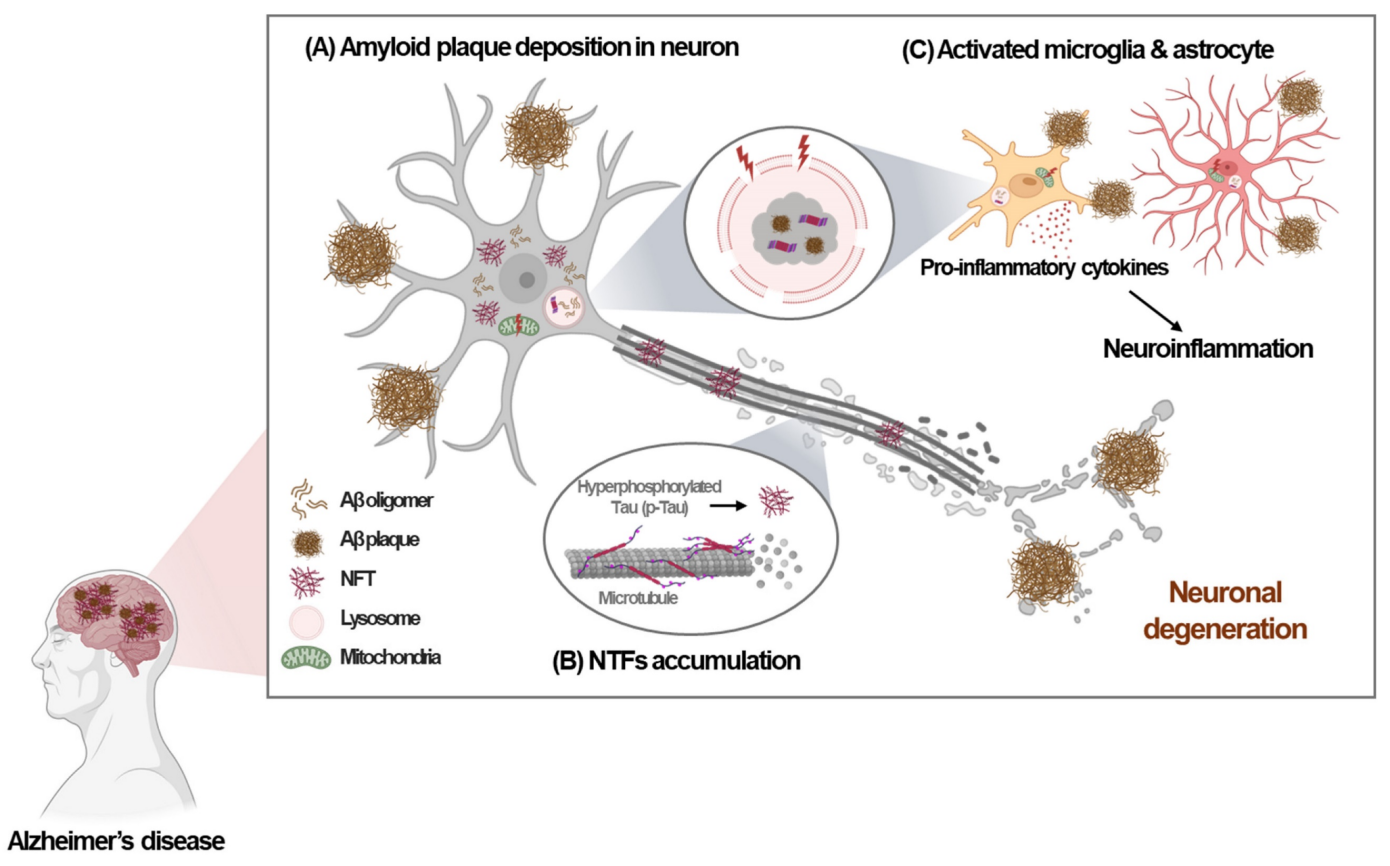
** Pathological hallmarks of Alzheimer's disease.** Alzheimer's disease (AD) is characterized by several key pathological features. (A) The deposition of amyloid-beta (Aβ) plaques. (B) Increased production of hyperphosphorylated Tau (p-Tau) leading to the formation and accumulation of neurofibrillary tangles (NFTs). (C) Activation or dysfunction of microglia and astrocyte induced by Aβ plaques or NFTs, leading to neuroinflammation and neuronal damage. These pathological features contribute to lysosomal and mitochondria stress, resulting in neuronal degeneration, further exacerbating AD pathology.

Alzheimer's disease (AD), which is characterized by amyloid deposition in the brain, memory loss, and cognitive dysfunction, is the most common cause of dementia worldwide. The pathological hallmarks of AD include the deposition of amyloid-beta (Aβ) aggregates, commonly known as amyloid plaques, and accumulation of hyperphosphorylated Tau (p-Tau) protein, resulting in neurofibrillary tangles (NFTs), along with widespread neuronal degeneration 1 (Fig. 1). Despite extensive characterization of the pathological features of AD, the precise mechanisms underlying AD pathogenesis remain incompletely understood.

Recent studies have shown the crucial role of the autophagy-lysosomal pathway in the clearance of Aβ and Tau aggregates, suggesting that dysfunction in this pathway could significantly contribute to AD development 2. Autophagy is a conserved cellular process responsible for degrading and recycling cytoplasmic components, including damaged organelles and protein aggregates via autophagosome formation followed by lysosomal degradation 3. Emerging evidence indicates that dysfunction of lysosome, effector organelle of autophagy, is causally associated with the accumulation of toxic protein aggregates and the progression of neurodegenerative diseases, including AD 2.

Previous studies focused on lysosomal dysfunction in AD have primarily examined lysosomal function in neurons. However, recent research has revealed the importance of the microglial lysosomal system in the pathogenesis of AD. Microglia are critical immune cells that play an important role in maintaining brain homeostasis. In AD, microglia may exert protective effects by clearing Aβ through phagocytosis and lysosomal degradation and by preventing the accumulation of toxic aggregates 4. However, if microglial reactions become chronic or dysfunctional due to genetic risk factors, they may contribute to neuroinflammation and neuronal damage, exacerbating AD pathology 4 (Fig. 1).

This review provides a comprehensive updated overview of the mechanisms of the lysosomal dysfunction implicated in AD pathogenesis not only in neurons but also in glial cells, and its effects on neurological deficit characterizing AD. Thus, our review might provide additional cutting-edge information regarding the role of lysosomal dysfunction in the pathogenesis of AD to the current reviews 5, 6. We additionally discuss the mechanism of lysosomal adaptation to the lysosomal dysfunction or stress associated with AD, and finally address pharmacological strategies that target lysosome for a potential therapeutic intervention. Modulating the autophagic flux and restoring lysosomal function may provide promising avenues for AD treatment.

## 1. The lysosome as an effector organelle in autophagy

Ubiquitin-proteasome system and autophagy-lysosome pathway are the two primary mechanisms responsible for protein degradation within cells. Ubiquitin-proteasome system primarily targets short-lived, soluble, or unfolded proteins 7, while autophagy-lysosome pathway preferentially degrades long-lived, amyloidogenic, or aggregated proteins 8. Autophagy-lysosome pathway is also essential for maintaining cellular homeostasis and nutrient balance 3.

Lysosomes are membrane-bound organelles enclosed by a lipid bilayer, containing more than 60 types of hydrolases that break down macromolecules within an acidic environment 9. This degradation process occurs in autophagy and phagocytosis, helping to ensure cellular homeostasis and support immune responses 9. Beyond their role in degradation, lysosomes are also critical for nutrient sensing and cellular signaling 9.

Transcription Factor EB (TFEB) serves as a master regulator of autophagy-lysosome pathway, responding to cellular stress by translocating to the nucleus and activating the transcription of autophagy and lysosomal genes 3, 10. Under nutrient-rich conditions, TFEB is phosphorylated and retained in the cytosol. However, under stress conditions, TFEB is dephosphorylated by calcineurin or other phosphatases, enabling its movement to the nucleus. Once in the nucleus, TFEB binds to coordinated lysosomal expression and regulatory (CLEAR) elements, promoting the expression of genes involved in autophagy and lysosomal biogenesis 11.

## 2. Dysregulation of lysosomal function in AD

Numerous studies have reported abnormal autophagic activity in AD, which may contribute to the failure to clear amyloidogenic or aggregate-prone proteins that are pathogenically linked to AD development 12, 13. Interestingly, an increased number of autophagosomes has been observed in the brain tissues of AD patients 14. This accumulation may result from diminished lysosomal function or impaired autophagosome-lysosomal fusion 15. Consistent with this, defective lysosomal function has been detected even before the formation of amyloid plaque 16, suggesting that lysosomal dysfunction may play a fundamental role in the accumulation of amyloid plaques and in the pathogenesis of AD.

### 2.1 Genes or proteins related to lysosomal function in AD

As strong evidence suggesting that lysosomal dysfunction plays a key role in the development of AD, several genes associated with early-onset AD have been linked to lysosomal dysfunction. Furthermore, a significant association has been observed between single nucleotide polymorphisms (SNPs) of various lysosomal genes and the development of sporadic AD.

One notable gene related to autophagy and lysosomal function in AD is the *mammalian target of rapamycin* (*mTOR*), a serine threonine kinase that negatively regulates autophagy. mTOR inhibits autophagy by phosphorylating key residues of TFEB and suppressing the unc-51-like autophagy-activating kinase (ULK1) complex 17. In AD patients, increased expression and phosphorylation of mTOR have been observed in the hippocampus, together with elevated levels of its downstream targets, such as *S6* kinase 1 (S6K1) and regulatory-associated protein of TOR (RAPTOR). These changes were correlated with cognitive impairment, linking mTOR to autophagy-lysosomal dysfunction in AD 18, 19. In 3xTg-AD mouse model, increased mTOR activity is observed in the hippocampus and cortex. This heightened activity is evident at both 6 and 12 months of age, corresponding with the presence of Aβ and Tau pathologies. The increase in mTOR signaling is marked by elevated levels of phosphorylated p70S6K, a downstream target of mTOR, in these brain regions 20 (Table 1).

Another gene of interest is *Nuclear Receptor Binding Factor 2* (*NRBF2*), which modulates autophagy by interacting with autophagy-related protein 14-like protein (*Atg14L*) and enhancing the kinase activity of the BECN1-PIK3C3 complex (VPS34) 21. In AD patients, NRBF2 activity was found to be reduced in the parahippocampal gyrus and hippocampus 22. In the hippocampus of 5xFAD AD mice, the expression levels of NRBF2 are reduced, indicating a potential link between decreased NRBF2 levels and AD pathology. Overexpression of NRBF2 in human mutant APP-overexpressing cells promotes the degradation of APP-CTFs and reduces levels of Aβ. Conversely, when NRBF2 is knocked out, there is an increase in APP-CTFs and Aβ, suggesting that lower NRBF2 levels may exacerbate AD-related processes 23. Additionally, overexpression of NRBF2 in 5xFAD mouse model led to a reduction in Aβ deposition and improvements in memory 22, suggesting that NRBF2 plays a role in regulating autophagy and lysosomal function related to Aβ clearance and neuronal health (Table 1).

A classic example of a lysosomal protein related to AD is Cathepsin D (CTSD), a lysosomal protease involved in degrading amyloid precursor protein (APP) and Tau. Mutations and polymorphisms in the *CTSD* gene, such as SNP rs17571, have been linked to an increased risk of AD, which is likely to be associated with its effect on Tau processing 24. Notably, the SNP rs17571 was associated with a higher risk of AD in male carriers, with no significant effect observed in women, suggesting a potential gender-specific effect 25. The deletion of CTSD in APP transgenic mice resulted in significant increases in both soluble and insoluble forms of Aβ, which forms intense intracellular aggregates, while extracellular Aβ deposition is not affected by CTSD deletion 26. Interestingly, CTSD KO mice develop significant tauopathy by about three weeks of age, characterized by the accumulation of hyperphosphorylated tau, which is more severe than in other mouse models of tauopathy, such as JNPL3, without the need for overexpression of human tau with disease-associated mutations 26 (Table 1).

Another lysosomal protease, Cathepsin E (CTSE), is also implicated in AD pathogenesis. Elevated levels of CTSE have been observed in the cortex of AD patients and in the hippocampus of 6-month-old APP knockin (KI) mice 27. Inhibition of CTSE improved memory function and reduced Aβ accumulation and neuroinflammation in APP KI mice 27, positioning CTSE as a potential therapeutic target for treatment of AD. CTSE has also been suggested to be a biomarker for familial amyloidotic polyneuropathy (FAP) 28 (Table 1).

Preseniln-1 (PS1), a component of the γ-secretase complex that catalyzes the intramembrane proteolysis of type I membrane proteins such as APP and Notch, is another key molecule linking lysosomal dysfunction and the development of AD 29. Mutations in the *PSEN1* gene, which encodes PS1, are the most common causes of familial AD 29. Recent studies have shown that PS1 regulates lysosomal function and autophagy by modulating vacuolar ATPase (v-ATPase) activity, lysosomal acidity, and lysosomal Ca^2+^ content 29, 30, as discussed in the following chapter (Table 1).

Another example of protein which could be associated with the development of AD and also regulates lysosomal function is progranulin. Mutations of *PGRN* encoding neuroprotective glycoprotein, progranulin, is linked to the development of neurodegenerative diseases such as frontotemporal dementia (FTD) or amyotrophic lateral sclerosis (ALS) 31. Progranulin regulates lysosomal function such as maturation of cathepsins and maintenance of the level of bis(monoacylglycerol)phosphate (BMP), a late endosome/lysosome-specific phospholipid 32, and *PGRN* knockout (KO) is associated with severe lysosomal dysfunction such as defective lysosomal acidification. Progranulin binding to Sortilin leads to endocytosis of progranulin and after delivery of progranulin to lysosome, Sortilin is cycled back to the plasma membrane 33. In addition to FTP and ALS, progranulin mutation can be a risk factor of AD 34. Furthermore, it has been reported that progranulin level in the cerebrospinal fluid (CSF) or serum could be a diagnostic or prognostic factor in AD 35, and *PRGN* gene delivery could ameliorate signs of AD in the Tg2576 mouse model of AD 36 (Table 1).

*Sortilin-related receptor 1* (*SORL1*) variant is also a risk gene for both rare genetic AD and common sporadic one. SORL1 plays a role in sorting and trafficking of APP between endosome, Golgi complex and lysosome. SORL1 binds and protects APP from amyloidogenic processing. SORL1 can also bind newly-formed Aβ and guide to the lysosome for degradation. Without SORL1, APP moves to late endosome where APP can be cleaved by secretase forming Aβ 37, which could explain the mechanism of AD associated with *SORL1* variants (Table 1).

*BIN1* encoding amphiphysin 2 has been identified as a locus of genetic risk to AD, and the second most significant susceptibility locus of sporadic AD after apolipoprotein E (APOE). BIN1 functions in membrane trafficking and endocytosis. In the absence of BIN1, BACE1 is no longer transported to the lysosome and degraded but instead accumulates in early endosomes, promoting Aβ generation 38. BIN1 K358R mice expressing a risk allele showed increased amyloid burden when crossed to 5xFAD mice which was accompanied by attenuated response of glial cells such as GFAP induction but not of microglial cells 39. BIN1 has been shown to interact with Tau. Tau PS19 BIN1 -KO mice exhibited more severe neurological deficit compared to PS19 control mice with increased Tau load in the somatosensory cortex but decreased Tau load in the hippocampus, suggesting region-specific effects of *BIN1* 40.

*PICALM* encoding phosphatidylinositol-binding clathrin assembly protein (PICALM) is a risk gene for sporadic AD identified in GWAS studies. PICALM senses membrane curvature and controls size, maturation and completion of clathrin-coated endocytic vesicles. PICALM plays an important role in clathrin-mediated endocytosis (CME) and also in autophagy through endocytosis of vesicle-associated membrane proteins (VAMPS) proteins and regulation of lysosomal function such as cathepsin processing 41, 42. PICALM has also been reported to rescue endocytic uptake defect mediated by APOE4 43. PICALM complexed with AP2 has been reported to crosslink LC3 with APP C-terminal fragment (APP-CTF) for autophagic degradation 44. The association of the *PICALM* rs3851179 allele with cortical thickness, as well as CSF levels of Aβ42 and p-Tau, has been reported in elderly subjects 45. Reduced endothelial PICALM was observed in the brains of AD patients, and this deficiency is associated with delayed Aβ clearance, exacerbating Aβ pathology 46.

### 2.2 Faulty acidification as a causative factor of lysosomal dysfunction in AD

**Figure 2:**
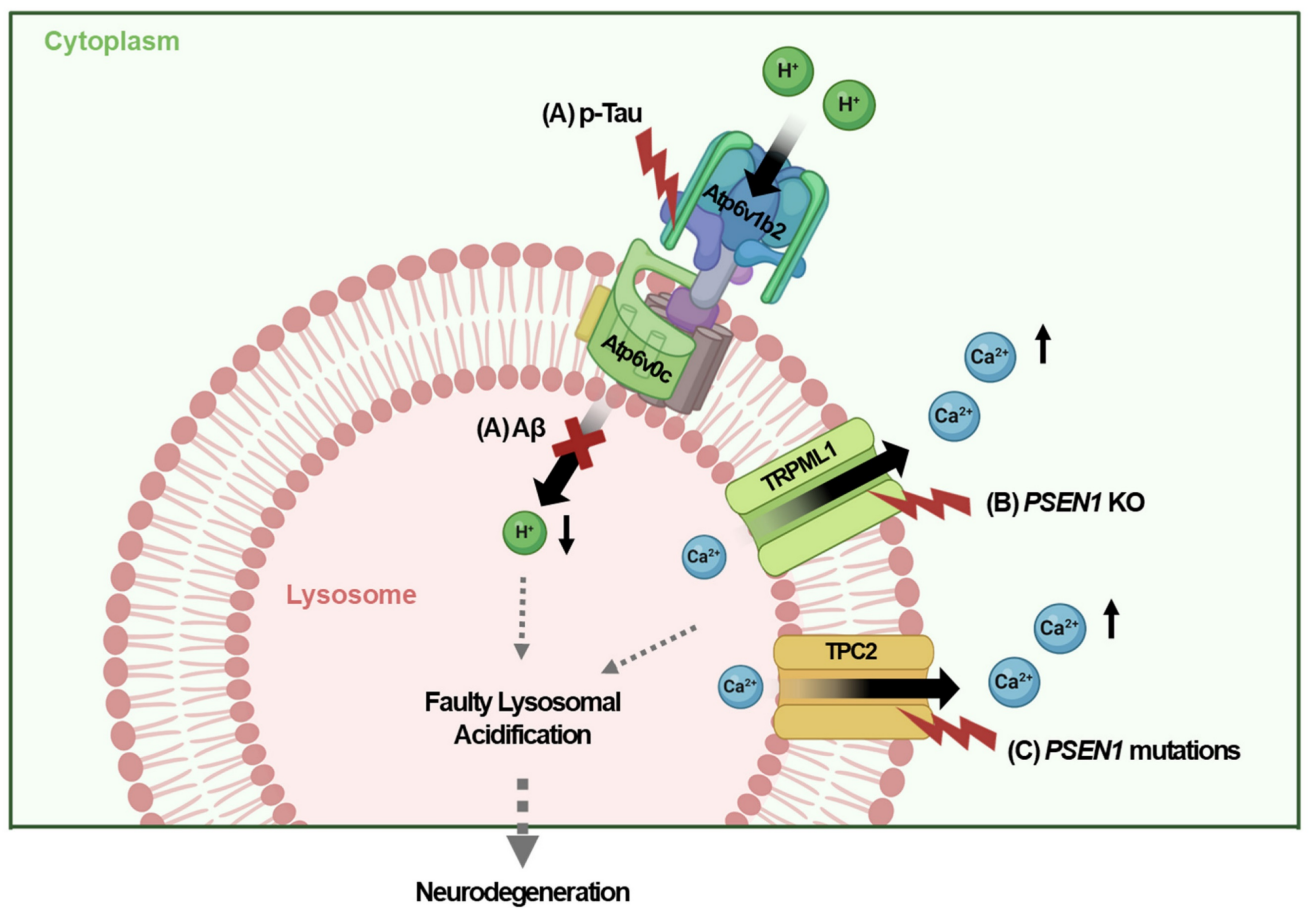
** Faulty lysosomal acidification in AD.** (A) Luminal and cytosolic subunits of the v-ATPase complex interact with internalized Aβ and cytosolic p-tau, impairing v-ATPase activity in neurons. (B) Deletion of *PSEN1* results in abnormal Ca^2+^ efflux via TRPML1, thereby increasing cytosolic Ca^2+^ levels in neurons. (C) Mutant *PSEN1* disturbs the lysosomal pH by enhancing Ca^2+^ release via TPC2, resulting in lysosomal acidification defect in neurons.

Recent studies have highlighted the crucial role of impaired lysosomal acidifications in the pathogenesis of AD. Proper lysosomal function depends on the acidic pH of 4.5-5, which is regulated by the v-ATPase that pumps protons into lysosomes via ATP hydrolysis 9. Dysfunction in v-ATPase disrupts lysosomal acidification, leading to impaired clearance of aggregate proteins, which contributes to AD. Notably, lysosomal acidification impairment has been observed early in the disease process, before neurodegeneration and the appearance of overt pathological changes 16. In Tg2576 AD mouse models, a significant decline in autolysosome acidification occurs prior to extracellular amyloid deposition, which is linked to reduced v-ATPase activity and the accumulation of Aβ and β-C-terminal fragments (CTFs) in enlarged, de-acidified autolysosomes 16. While Aβ fibril and Tau aggregates would accumulate in dysfunctional lysosome with impaired proteolytic activity, both Aβ and Tau been identified as key inducers of lysosomal dysfunction in AD, culminating in the feed-forward mechanism between lysosomal dysfunction and Aβ/ Tau accumulation. For instance, the luminal subunit Atp6v0c of v-ATPase binds to internalized Aβ, while the cytosolic subunit Atp6v1b2 interacts with p-Tau 47. These interactions disrupt v-ATPase function, leading to endolysosomal dysfunction, disrupted endolysosomal integrity, and neurotoxicity or neurodegeneration 47 (Fig. 2A).

Genetic mutations associated with AD also impact lysosomal dysfunction and acidification. For example, the genetic deletion or mutation of *PSEN1,* a gene implicated in AD, has been reported to disrupt lysosomal acidification and proteolysis due to defective v-ATPase maturation, which impairs autophagy 29, 30. Elevated lysosomal pH in *PSEN1* KO neuronal cells affects transient receptor potential mucolipin 1 (TRPML1) activity, leading to abnormal Ca^2+^ efflux from lysosomes into the cytosol 30 (Fig. 2B). In contrast to these results suggesting the primary role of disturbed v-ATPase maturation accompanied by subsequent alteration of Ca^2+^ efflux, primary role of abnormal Ca^2+^ efflux in *PSEN1* mutations has also been reported 47. Recent research has also suggested the role of lysosomal Ca^2+^ channels other than TRPML1 in the context of *PSEN1* mutations 48. Mutant *PSEN1* has been shown to increase Ca^2+^ release through two-pore channel 2 (TPC2), resulting in lysosomal alkalization and impaired lysosome function. Inhibition of TPC2 can restore Ca^2+^ homeostasis in lysosomes, normalizing both pH and function 49 (Fig. 2C). Thus, the regulation of lysosomal Ca^2+^ is crucial not only for the expression of autophagy genes and lysosomal biogenesis via TFEB activation but also for maintaining lysosomal acidity, which is essential for neuronal function and the pathogenesis of AD 50, 51.

Additionally, dysregulation of other organelles that interact with lysosomes can contribute to defective lysosomal function and acidification. For instance, abnormal Ca^2+^ flux through the endoplasmic reticulum (ER)-resident ryanodine receptor (RyR) has been shown to impair lysosomal acidification by reducing the expression of v-ATPase subunits, such as V0a1. This leads to lysosome deacidification and disrupted proteolytic activity in 3xTg AD mouse models and human neurons derived from AD patients 52. Restoring ER Ca^2+^ balance can reverse lysosomal dysfunction in 3xTg AD mouse models 52. Therefore, ER-lysosome Ca^2+^ coupling is crucial for lysosomal acidification, vesicular trafficking, nutrient sensing, and the processes of autophagy and mitophagy 53, 54. Lysosomal acidification and Ca²⁺ regulation are key aspects of cell physiology in the pathogenesis of AD. However, further research is necessary to develop therapeutics that target this pathway.

### 2.3 Other mechanisms of lysosomal dysfunction in AD

Lysosomal dysfunction in AD involves several mechanisms beyond faulty lysosomal acidification, including defective autophagy-lysosome fusion, release of lysosomal hydrolases, and disruptions associated with APOE4. These mechanisms highlight the broad roles of lysosomal dysfunction in AD pathogenesis.

#### 2.3.1 Defective autophagosome-lysosome fusion

Fusion between autophagosome and lysosomes is an essential event in autophagy 55. Defective fusion between them or premature fusion of unsealed autophagosomes can impair the degradation of cargos or autophagosome inner membrane 56. The autophagy-related protein 8 (ATG8) family such as gamma-aminobutyric acid receptor-associated protein (GABARAP) plays a vital role in regulating autophagosome-lysosome fusion by recruiting pleckstrin homology domain containing protein family member 1 (PLEKHM1) to autophagosomes. This process is facilitated by soluble *N*-ethylmaleimide-sensitive factor attachment protein receptor SNARE proteins, such as STX17, SNAP29, and lysosome-localized VAMP8 or VAMP7, with YKT6 acting independently of STX17 via SNAP29 57. Other regulators, like RAB GTPases (RAB7A, RAB33B, and RAB2A), and effectors such as PLEKHM1, are also involved in this fusion process 58.

**Figure 3:**
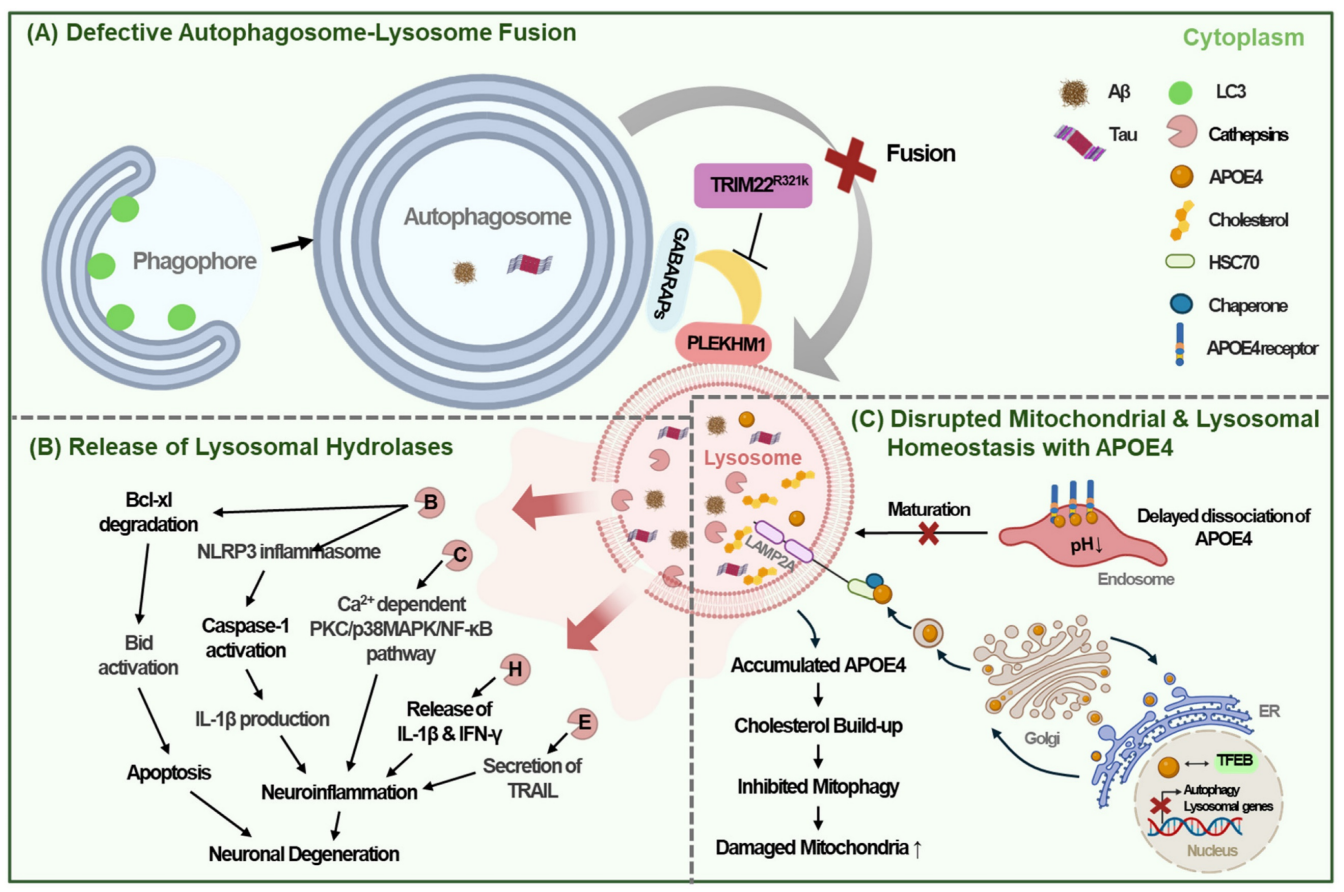
** Autophagy lysosomal dysfunction in AD.** (A) The TRIM22 R321K variant inhibits the association between GABARAPs and PLEKHM1, thereby impeding autophagosome-lysosome fusion and interfering with intracellular autophagic clearance. (B) Release of intra-lysosomal hydrolases such as cathepsin B, C, H, E into the cytoplasm caused by lysosome membrane permeabilization impairs intra-lysosomal enzyme activity and promotes neuroinflammation, leading to neuronal degeneration. (C) APOE4 is transported to the lysosome by LAMP2A from post-Golgi compartment and accumulates in lysosome. Delayed dissociation of APOE4 from its receptors in the endosome prevents endolysosomal trafficking. Accumulated APOE4 and resultant cholesterol build-up in lysosome disturb autophagy flux and inhibit mitophagy. APOE4 also impairs TFEB-mediated lysosomal biogenesis and autophagy.

Tripartite motif-containing 22 (TRIM22) further aids in autophagosome-lysosome fusion by associating GABARAP family proteins with PLEKHM1. TRIM22 interacts with GABARAP proteins rather than LC3 family proteins, indicating its specialized role in the fusion process rather than in autophagosome formation 15, 58. The TRIM22 R321K variant disrupts the association between PLEKHM1 and GABARAPs, potentially hindering their fusion and subsequent autophagic clearance 15. This variant is associated with early-onset familial AD, highlighting the critical importance of TRIM22-mediated autophagosome-lysosomal fusion in the clearance of amyloid or protein aggregates and presenting potential therapeutic targets for addressing autophagic or lysosomal dysfunction in AD 15 (Fig. 3A).

#### 2.3.2 Release of lysosomal hydrolases

The lysosomal system comprises over 80 hydrolases, including proteases, nucleases, phosphatases, sulfatases, lipases, and glycosidases, all essential for maintaining proper lysosomal function. Lysosomal cathepsins are the most abundant lysosomal proteases and include cysteine (cathepsins B, C, F, H, K, L, O, S, V, W, and X), aspartic (CTSD and CTSE), and serine (cathepsin G) proteases 59. To prevent leakage of lysosomal enzymes and damage to cytosolic constituents and also to protect lysosomal acidity, maintenance of lysosomal membrane integrity is vital. Saposins (SAPs) are lysosome-restricted proteases lysosome activator/lysosomal restricted proteins that are involved in cathepsin maturation and progranulin transport. Elevated levels of SAPs and LAMP1 were observed around amyloid plaques in the brains of APP KI, 5xFAD and APP/PS1 mice in the early stages of the disease, potentially contributing to lysosomal membrane leakage and the subsequent hydrolase loss 60.

Regarding the consequences of lysosomal membrane leakage, lysosome membrane permeabilization can release intracellular components, such as cathepsins, into the cytoplasm, leading to lysosomal-dependent cell death, which may contribute to neuronal loss in AD 61. For instance, cytosolic cathepsin B (CTSB) initiates a proteolytic cascade that results in apoptosis by degrading the apoptosis-preventing protein Bcl-xL and activating the apoptosis-inducing protein Bid 62. Moreover, the release of cathepsins can trigger neuroinflammation by inducing the release of inflammatory cytokines from microglia. Cathepsin C, for example, exacerbates neuroinflammation by inducing the M1 polarization of microglia through activation of the Ca^2+^ dependent PKC/p38MAPK/NF-κB pathway 63. Similarly, cathepsin H induces neuroinflammation by releasing interleukin-1β* (IL*-*1β*) and interferon-γ (IFN-γ), leading to neuronal death 64. Furthermore, lysosomal CTSB leakage promotes the assembly of the NLR family pyrin domain containing 3 (NLRP3) inflammasome, which triggers IL-1β production and caspase-1 activation in macrophages 65. Microglial CTSE also contributes to neuroinflammation by enhancing the secretion of TNF-related apoptosis-inducing ligand (TRAIL), which further increased both NF-κB-dependent microglial neuroinflammation and BACE-mediated neuronal Aβ production 27 (Fig. 3B).

#### 2.3.3 Disrupted mitochondrial and lysosomal homeostasis associated with APOE4

*APOE4* is a major genetic risk factor for AD and associated with lysosomal dysfunction and abnormal nutrient metabolism. Carriers of the *APOE4* allele exhibit increased aerobic glycolysis and impaired oxygen consumption in the brain 66, 67, which is linked to altered glucose metabolism, dysregulated lipid homeostasis, and lysosomal/autophagosomal impairments in neurons and astrocytes 68.

Individuals carrying the *APOE4* allele have APOE4 transported from the post-Golgi compartment to the endolysosome via lysosome-associated membrane glycoprotein 2A (LAMP2A)-mediated; chaperon-mediated autophagy; consequently, APOE4 accumulates in the lysosomes 69.

Dissociation of APOE4 from its receptors in the endosome has been reported to be delayed due to high isoelectric point, which may impair endolysosomal trafficking through progressive acidification 43, 70. In the lysosomes of glial cells or human induced pluripotent stem cells (iPSCs) with *APOE4* variants, there is excessive cholesterol accumulation due to upregulated de novo synthesis, despite elevated intracellular cholesterol due to sequestration in lysosomes 71, 72. This excessive lysosomal cholesterol can inhibit autophagic flux and mitophagy, leading to mitochondrial dysfunction 73. Reducing lysosomal cholesterol accumulation has been shown to restore both autophagy and mitochondrial homeostasis, highlighting a critical role of elevated lysosomal cholesterol in impaired lysosomal and mitochondrial function 73 (Fig. 3C).

Consistent with these findings, astrocytes from AD patients exhibit reduced mitochondrial respiration, increased mitochondrial reactive oxygen species (ROS), circular mitochondria, impaired mitophagy, and the accumulation of dysfunctional mitochondria 73. Additionally, *APOE4* has been shown to inhibit autophagy gene expression by directly binding to the CLEAR sites in the promoters of TFEB target genes 74 (Fig. 3C). Importance of microglial endolysosomal system in APOE4-mediated aggravation of AD has also been reported. A recent paper reported that APOE, particularly APOE4, aggregates primarily in endolysosome of microglia which was aggravated by lysosomal dysfunction and could initiate amyloid seeding 75. Intriguingly, enhanced binding of APOE4 with low-density lipoprotein receptor (LDLR) was also reported to contribute to the increased endolysosomal delivery of LDLR-associated cholesterol esterified with polyunsaturated fatty acids inducing lipofuscin accumulation 76. Furthermore, an *APOE* R136S mutation (APOE Christchurch, *APOEch*) which has protective effect against AD development could downregulate APOE binding to LDLR reducing lipid burden which might explain the mechanism of clinical benefits of *APOEch* mutation 76. These findings were observed not only in neurons but also in astrocytes and microglial cells, supporting effect of APOE in a wide variety of cells in the CNS participating in the pathogenesis of AD 76.

While both mutations of presenilin and APOE4 can affect lysosomal function in association with the development of AD, their interaction is far from clear. *PSEN1/2* mutations and *APOE4* are associated with different types of AD in that *PSEN1/2* mutations are the most prevalent causes of genetic AD, and *APOE4* is the strongest risk factor for sporadic AD. However, they can interact with each other in either genetic or sporadic AD. For instance, APOE4 has been reported to inhibit γ-secretase activity of presenilin through direct binding. *APOE4* KO was associated with increased Aβ40 level and γ-secretase activity. In contrast, *APOE4* KI resulted in lower Aβ40 level and γ-secretase activity but increased Aβ42/Aβ40 ratio, which might be related to the increased Aβ42/Aβ40 ratio in the brain of AD 77. An intriguing finding related to the interaction between *APOE* and *PSEN* mutations is amelioration of AD phenotypes in patients with E280A *PSEN1* mutation by *APOE3 R136S* mutation (*APOE3ch*). *APOE3ch* has been found to impair APOE binding to heparan sulfate proteoglycan (HSPG) and LDLR-related protein 1 (LRP1) involved in neuronal Tau uptake and spreading, which can explain reduced Tau accumulation and spreading in patients with homozygous *APOE3ch* mutation 78. Recent papers reported enhanced microglial lysosomal activity and increased microglial phagocytosis of Tau fibrils followed through degradation by *APOE3ch* mutation 79, supporting the effect of APOE3 on lysosomal function. Furthermore, *APOE3ch* mutation restored lysosomal enlargement observed in astrocytes of a mouse model expressing a mutant Tau and APOE4 80, supporting the effect of APOE4 on lysosomal function and its reversal by APOE3 R136S.

Despite these results suggesting the relationship between APOE4 and lysosomal dysfunction associated with AD, the detailed mechanism of endolysosomal dysfunction by APOE4 and differential effect of *APOE3* vs. *APOE4* on the pathogenesis of AD are still far from clear and warrants further studies 69, 81.

While these observations suggest a link between APOE4 and lysosomal dysfunction associated with AD, the precise mechanisms of endolysosomal dysfunction caused by APOE4 remain unclear and require further investigation.

## 3. Lysosomal dysfunction in microglia associated with AD

Autophagy and lysosomal function are crucial not only in neuronal cells but also in glial cells, particularly in microglia, which are key components of the innate immune system in the central nervous system (CNS). Microglia are responsible for engulfing and degrading brain-derived products, including apoptotic cells, synapses, myelin, and Aβ aggregates, thereby maintaining physiological function and cellular homeostasis in the CNS 82. Under the pathological conditions, defective lysosomal acidification in microglia contributes to neuroinflammation and neurodegeneration, exacerbating inflammatory cytokine production through mechanisms such as altered polarization and lysosomal membrane permeabilization 82.

In AD, microglia surrounding amyloid plaques exhibit disrupted lysosomal function caused by the mislocalization of the CIC-7 transporter. This mislocalization is linked to low levels of osteoclastogenesis-associated transmembrane protein 1 in quiescent microglia, impairing the degradation of Aβ. Without proper CIC-7 function, lysosomal acidification is impaired and negatively affects Aβ degradation 4. Additionally, the dysregulation of TFEB has been observed in microglia associated with AD. Microglia with *PSEN1 S367A* mutations exhibit decreased *TFEB* mRNA levels, leading to impaired autophagy and inability to degrade Aβ 83. Impaired lysosomal function in microglia reduces both phagocytic and autophagic capabilities, preventing the clearance of damaged organelles, such as mitochondria, myelin debris, and toxic protein aggregates. As a result, it promotes neuroinflammation, neuronal death, and neurodegeneration in the CNS 84-86. Lysosomal dysfunction in microglia can lead to the accumulation of Aβ due to defective clearance, thereby exacerbating AD pathology 86. The importance of microglial lysosomal function in the pathogenesis of AD is strongly supported by the localization of AD-risk gene variants, such as *BIN1*, *APOE* and *PICALM,* in microglia-specific enhancer-promoter regions 87. In particular, the effects of APOE4 on the endolysosomal system of microglia through aggregation facilitating Aβ seeding 84 or increased lipid burden leading to aggravated inflammation 76 suggest the critical role of endolysosomal function in microglia as an important target of APOE4 75, as mentioned earlier.

Aβ deposition in microglia can also trigger neuroinflammatory responses, through mechanisms such as the activation of the NLRP3-dependent inflammasome due to extracellular Aβ. The relevance of microglial inflammation or inflammasome activation in AD is further supported by studies showing that switching microglia to an anti-inflammatory state through NLRP3 inflammasome KO prevents memory loss in APP/PS1 AD mice 88. Furthermore, microglial-specific deletion of *Autophagy-related 7* (*ATG7*), a key gene for autophagosome biogenesis, results in microglial transition to a pro-inflammatory state and inflammasome activation *in vitro*, which in turn enhances intraneuronal Tau pathology and its spread 89. Aβ-mediated inflammasome activation could be mediated by protein kinase AMP-activated catalytic subunit alpha 1 (PRKAA1) pathway 88, potentially contributing to the disease process through cell-to-cell propagation. For example, apoptosis-associated speck-like protein containing a CARD specks, a key component of inflammasome complex, released from microglia can bind to Aβ, forming oligomers and aggregates that promote cross-seeding 90. Similarly, Tau proteins or aggregates activate inflammasomes 91. Tau phosphorylation can be induced by IL-1β-dependent Tau kinases, forming a feedback loop between inflammasome activation and Tau pathology 92. Accentuated NLRP3 inflammasome activation in microglia with autophagy insufficiency or lysosomal dysfunction could arise from the accumulation of Aβ or Tau, as well as from the impaired clearance of the NLRP3 inflammasome complex itself by microglial autophagy 93, 94.

These findings underscore the critical role of the microglial autophagy-lysosomal system in modulating Aβ clearance, Aβ-dependent inflammatory responses, and the overall development of AD. As a corollary, enhancing microglial lysosomal function may have the potential to ameliorate AD-related neuroinflammation (Kim *et al.*, unpublished data).

## 4. Lysosomal Stress Response (LSR) in AD

The lysosomal stress response (LSR) is crucial for counteracting lysosomal damage and maintaining cellular integrity in AD. Cells have evolved several mechanisms to address lysosomal stress, including repair, lysophagy, replacement, and autophagic lysosomal reformation (ALR), making these pathways promising therapeutic targets for AD 95.

### 4.1 Lysosomal Repair

**Figure 4:**
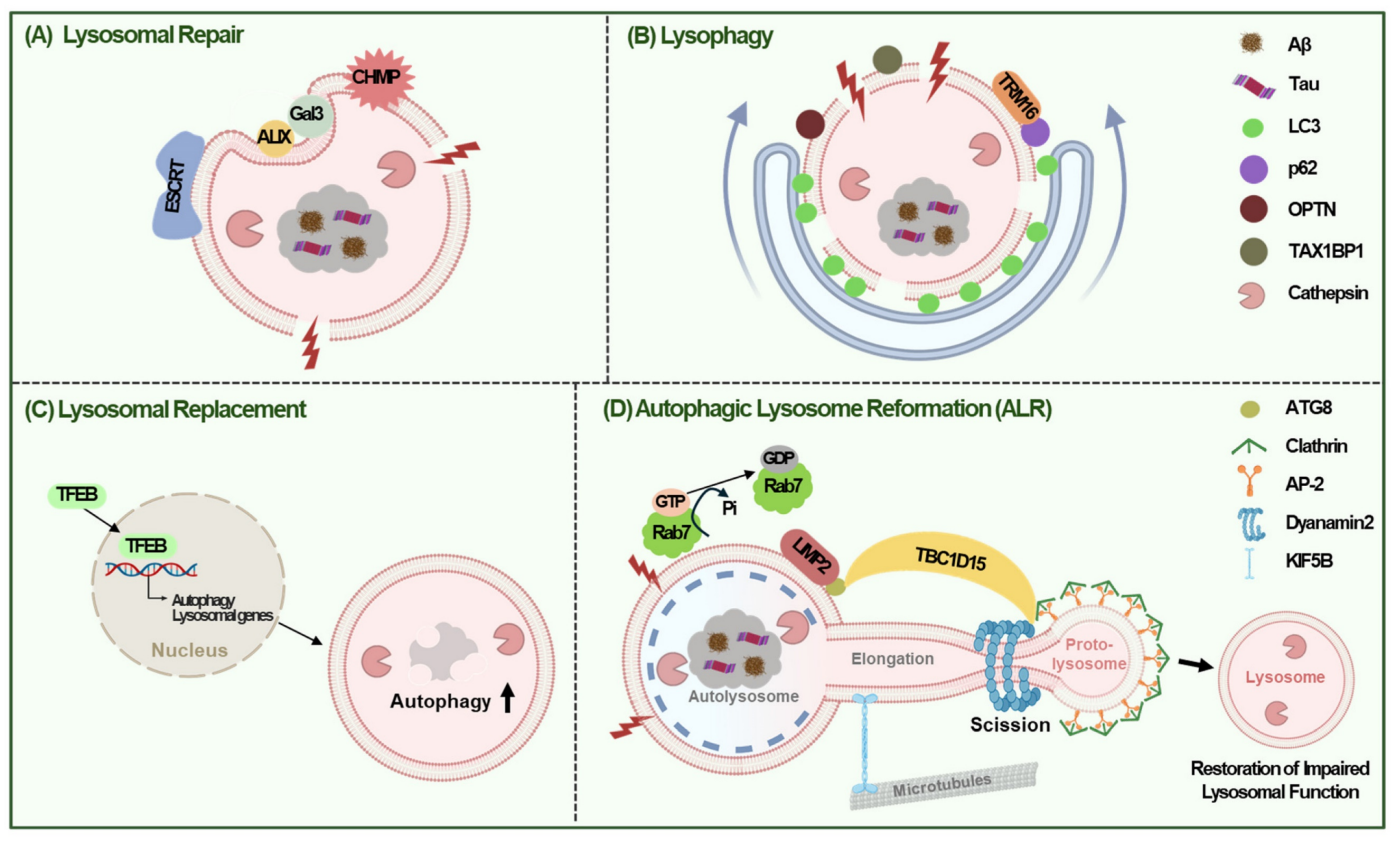
** Lysosomal Stress Response (LSR) in AD.** (A) Small disruptions in lysosomal membrane integrity can trigger lysosomal repair mechanisms that facilitate membrane sealing via the recruitment of ESCRT machinery and ALIX proteins, resulting in their rapid localization to the lysosome membrane when the lysosomal membrane is ruptured. (B) If this disruption is too severe to repair, lysosomes are targeted for self-degradation via lysophagy, a selective autophagy process. TRIM16 overexpression can enhance lysophagy by recruiting LC3, p62, OPTN, and TAX1BP1 to damaged lysosomes, thus inhibiting the high-glucose-induced accumulation of Aβ and tau. (C) Lysosome replacement involves the creation of new lysosomes to replace those that are damaged or lost, thereby ensuring the cell can continue to break down and recycle materials effectively. Lysosomal replacement is triggered by lysosome stress, and TFEB is a key regulator in lysosome regeneration by promoting lysosomal function. (D) ALR might also help restore impaired lysosomal function by lysosomal damage. During ALR, LIMP2 binds to ATG8 and then recruits TBC1D15, providing scaffold for assembly of ALR machinery. TBC1D15 hydrolyzes Rab7-GTP for segregation of damaged lysosome and Dynamin2-dependent scission of lysosomal tubule.

Minor disruptions in lysosomal membrane integrity activate repair mechanisms involving the endosomal sorting complex required for transport (ESCRT) 96, 97 and heat shock protein 70 (HSP70) 98. Proteins such as ESCRT-I, ESCRT-III, and ALG-2-interacting protein X (ALIX) are rapidly recruited to the ruptured lysosomal membranes to facilitate repair 99. Essential ESCRT components, such as Hrs and Tsg101, are required for lysosomal targeting of APP, and their reduction results in increased intracellular Aβ and decreased Aβ secretion 100. The knockdown of ESCRT components, including charged multivesicular body proteins (CHMP6, CHMP2A, and CHMP2B), promotes Tau aggregation 101. Furthermore, defects in the ESCRT machinery are associated with both sporadic and familial AD 99, suggesting the role of lysosomal recovery via the ESCRT pathway in protecting against Aβ- or Tau-mediated damage in the development of AD. Severe lysosomal damage triggers lysophagy, a selective autophagic process that degrade damaged lysosomes 98, 102 (Fig. 4A).

### 4.2 Lysophagy

When lysosomal repair through ESCRT pathway fails, lysosomes are tagged with ubiquitin and selective autophagy of lysosome called 'lysophagy' is initiated (66). During lysophagy, glycosylated proteins on damaged lysosomes are recognized by galectins, which recruit autophagy receptors like p62, Optineurin (OPTN), or Tax1 binding protein 1 (TAX1BP1) after ubiquitination 103. These receptors facilitate the engulfment of ubiquitinated lysosomes into autophagosomes, which then fuse with intact lysosomes for degradation. For example, galectin 3 recruits tripartite motif-containing 16 (TRIM16), while galectin 8 binds to nuclear dot protein 52 (NDP52), signaling lysosomal damage to mTORC1 and AMPK, thereby inducing autophagy 93, 104. Overexpression of TRIM16 enhances lysophagy by recruiting LC3, p62, and ubiquitin to damaged lysosomes, effectively reducing Aβ and p-Tau accumulation 105 (Fig. 4B).

### 4.3 Lysosomal Replacement

Lysosomal replacement involves creation of new lysosomes to replace damaged ones. This process requires production and endocytosis of new lysosomal proteins, which are triggered by lysosomal stress 3, 10. TFEB plays a critical role in promoting function and regeneration of lysosome by interacting with the CLEAR elements in lysosomal gene promoters, especially when mTOR is inhibited 106 (Fig. 4C).

### 4.4 Autophagic Lysosomal Reformation (ALR)

Besides the three mechanisms discussed above, ALR might also contribute to the lysosomal regeneration in response to lysosomal damage 107. ALR is characterized by extrusion of proto-lysosome from autolysosome and its maturation into functional lysosome, which can help restore impaired lysosomal function by lysosomal injury 107. In ALR following lysosomal injury, LIMP2, a lysosomal integral membrane protein-2 was found to bind Atg8 and then recruit TBC1 domain family member 15 (TBC1D15), which provides scaffold for assembly of ALR machinery. This process comprises hydrolysis of Rab7-GTP for segregation of damaged lysosome and Dynamin2-dependent but TFEB-independent scission of lysosomal tubule 107 (Fig. 4D).

These LSR mechanisms are essential for maintaining cellular homeostasis against lysosomal stress and offer potential therapeutic strategies for mitigating lysosomal dysfunction in AD.

## 5. TFEB as a target of therapeutic approaches enhancing autophagy-lysosome pathway

Several studies have reported that TFEB dysregulation is associated with AD, consistent with the role of lysosomal dysfunction in AD pathogenesis. For example, reduced nuclear TFEB levels have been observed in the brains of AD patients or aged experimental animals 109, 110. In contrast, increased expression of TFEB downstream genes has been noted in the brains of patients with AD. Although *TFEB* mRNA expression remained unchanged in cortical neurons, it is increased in the hippocampal region, likely due to its expression in glial cells. In contrast, the mRNA expression of *TFE3*, another member of the MiT family of bHLH-leucine zipper transcription factors alongside TFEB, has been shown to increase in both cortical and hippocampal regions in the brains of AD patients 18. This discrepancy may reflect variations in the disease stages studied, as TFEB-mediated adaptive changes to lysosomal dysfunction or stress in AD may fluctuate over the course of disease progression. However, these adaptive changes may not be sufficient to fully counteract the lysosomal dysfunction or reverse the disease course. Reinforcing these adaptive changes may provide an opportunity to halt or even reverse AD progression, making TFEB a promising target for these approaches.

## 6. Autophagy and lysosomal enhancers targeting TFEB in AD

Given the central role of dysregulated autophagy and lysosomal function in AD pathogenesis, various pharmacological strategies have been developed to target this pathway 5. These strategies focus on enhancing autophagy initiation, promoting lysosomal biogenesis, and activating TFEB-mediated transcriptional programs 110. Below are several pharmacological agents that have shown promise in preclinical models and are being evaluated for their potential therapeutic benefits in AD (Table 2).

Atractylenolide III, Ferulic acid and Paeoniflorin, either alone or in combination, counteract lipopolysaccharide (LPS)-induced autophagy inhibition and neuroinflammation. They enhance the expression of autophagy-related proteins including AMP-activated protein kinase (AMPK), ULK1, and TFEB in BV2 microglial cells, leading to improved autophagy and reduced neuroinflammation 111, 112. Caudatin activates peroxisome proliferator-activated receptor α (PPARα), which enhances lysosomal degradation of Aβ and p-Tau aggregates. This reduces the onset of AD and improves cognitive dysfunction in 3xTg-AD model mice 113. Celastrol promotes autophagy and lysosomal biogenesis by activating TFEB through mTORC1 inhibition. It ameliorates Tau pathology in P301S AD model mice 114, 115. Corynoxine, a natural autophagy enhancer, activates TFEB/TFE3 via inhibitions of the AKT/mTOR pathway and TRPML1-mediated Ca^2+^ release. This results in increased neuronal autophagy and lysosomal biogenesis, reduced APP-CTF levels, and improved cognitive function in 5xFAD AD mice 116. Crocetin stimulates autophagy by activating the serine/threonine kinase 11 (STK11/LKB1)-mediated AMPK pathway. It effectively crosses the blood-brain barrier, induces autophagy specifically in the hippocampus, reduces Aβ levels and neuroinflammation, and enhances memory function in 5xFAD AD mice 117. Curcumin-analog C1 activates TFEB, leading to enhanced autophagy and lysosomal activity. It reduces levels of APP, CTF-β/α, Aβ, and Tau aggregates, and improves cognitive function in P301S, 5xFAD and 3xTg AD mice models 118. Hederagenin upregulates TFEB and promotes the expression of autophagy-related proteins like LC3II/LC3I and LAMP2. This enhancement of autophagy and Aβ degradation alleviates cognitive impairment in APP/PS1 AD mice 119. Ibudilast inhibits mTORC1 activity and enhances TFEB-mediated lysosomal biogenesis and autophagy. This results in improved cognitive function and clearance of Aβ/Tau in 344-AD rats 120. Latrepirdine decreased mTOR phosphorylation and stimulated Atg5-dependent autophagy, reducing intracellular levels of Aβ. In TgCRND8 mice expressing a double mutant form of APP 695 (KM670/671NL+V717F) under the control of the PrP gene promoter, latrepirdine enhances autophagy, reduces Aβ deposition, and prevents further decline in behavioral abnormalities 121. LH2-051 enhances TFEB activation and lysosome biogenesis by inhibiting dopamine transporters (DAT). This improves cognitive function and Aβ clearance in APP/PS1 AD mice 122. ML246 is a small-molecule autophagy inducer that exerts BECN1-dependent protective effects. It enhances Aβ removal and memory in 5xFAD AD mice 123. ML-SA1, an agonist of the essential endosomal-lysosomal Ca2+ channel TRPML1, has been shown to reverse several key abnormalities in the endosomal-autophagic-lysosomal pathway in primary neurons. This effect is similar to those observed in AD-affected neurons after PIKfyve inhibition 124. Oleoylethanolamide enhances microglial Aβ clearance and improves cognitive function in 5xFAD AD mice by promoting TFEB lysosomal function in a PPARα-dependent manner, independent of mTORC1 125. Spermidine activates autophagy by promoting the hypusination of eukaryotic translation initiation factor 5A-1 (eIF5A), which upregulates TFEB translation 126. Treatment with spermidine in APP/PS1 AD mouse model activates autophagy, particularly in microglia, leading to reduced Aβ and neuroinflammation 127. Thonningianin A triggers autophagy via the AMPK/ULK1 and Raf/MEK/ERK pathways and promotes the degradation of NLRP3 inflammasome in microglial cells treated with Aβ 128. In APP/PS1 AD mice, it improves cognitive function, reduces Aβ accumulation, and decreases neuronal apoptosis 128. Trehalose, a natural disaccharide, activates the transcription of autophagy genes and TFEB. This activation occurs through low-grade lysosomal stress in an mTOR-independent, forkhead box protein O1 (FOXO1)-dependent manner, leading to a reduction in amyloid-β deposition in the hippocampus of APP/PS1 AD mice 129. Uric acid activates microglial autophagy and increases levels of autophagy-related proteins, including the LC3II/I ratio, Beclin-1, and LAMP1. It also helps alleviate memory decline and promotes Aβ degradation in APP/PS1 mouse model of AD 130 (Table 2).

## Conclusion and prospect

AD is a devastating neurodegenerative disorder marked by the accumulation of Aβ plaques and Tau tangles, leading to progressive cognitive decline and memory loss. Recent studies on the autophagy-lysosome pathway have revealed promising therapeutic strategies by targeting lysosomal dysfunction, a critical factor in AD pathogenesis.

Lysosomal dysfunction, especially due to faulty acidification, contributes significantly to the accumulation of Aβ and Tau, key factors in AD. Reduced lysosomal acidification, often caused by impaired v-ATPase activity, leads to the buildup of these toxic proteins, exacerbating the disease. Genetic mutations, such as those in *PSEN1*, further exacerbate lysosomal dysfunction by affecting v-ATPase maturation and Ca^2+^ homeostasis. Additionally, defective autophagy-lysosome fusion and the release of lysosomal hydrolases contribute to neurodegeneration and neuroinflammation. The APOE4 allele, a major genetic risk factor for AD, is associated with abnormal cholesterol accumulation within lysosomes, disrupting both autophagic and mitochondrial functions and accelerating disease progression.

In AD, lysosomal dysfunction is further aggravated by Aβ or Tau oligomer accumulation, which impairs essential lysosomal functions. Aging, a major risk factor for sporadic AD, exacerbates lysosomal dysfunction by reducing autophagic activity, leading to a buildup of toxic aggregates that overwhelm the lysosomal system. This cascade creates a vicious cycle that is further complicated by oxidative damage, altered enzymatic activity, and compromised lysosomal membrane integrity, ultimately resulting in neuronal toxicity and cell death. In AD, there is disrupted lysosomal acidification and Ca²⁺ regulation, leading to impaired autophagic flux. Impaired autophagy and lysosomal function also contribute to neuronal cell death through necroptosis 131, with autophagy deficits causing the accumulation of proteins such as receptor-interacting protein kinase-1 (RIPK1) and receptor-interacting protein kinase-3 (RIPK3) that are typically degraded by lysosomes 132. Elevated levels of RIPK1 and RIPK3 can trigger necroptosis, a programmed cell death pathway, and increased tumor necrosis factor receptor-1 (TNFR1) signaling in AD further promotes necroptosis through the RIPK1/RIPK3/MLKL pathway 131.

To counteract lysosomal dysfunction, the LSR plays a crucial role in maintaining cellular integrity by addressing lysosomal damage via mechanisms such as repair, lysophagy, replacement, and ALR. Repair processes, including those facilitated by the ESCRT machinery, are associated with intracellular Aβ and Tau aggregation when defective. Lysophagy, which is mediated by proteins such as galectins and autophagy receptors, selectively degrades damaged lysosomes. Lysosomal replacement regulated by TFEB promotes the generation of new lysosomes, essential for restoring lysosomal function. While ALR contributes to lysosome regeneration through membrane remodeling, independently of TFEB. These pathways are vital for maintaining cellular homeostasis and may present potential therapeutic targets for AD.

Given the crucial role of the dysregulated autophagy-lysosome pathway system, particularly, lysosomal dysfunction in AD development, targeting the autophagy-lysosome pathway and modulating lysosomal stress offers significant therapeutic potential through addressing the fundamental mechanisms underlying AD, enhancing the clearance of pathological proteins, and reducing neuroinflammation. TFEB has emerged as a promising therapeutic target for treating neurodegenerative disorders. However, there are several challenges and safety concerns associated with the development of TFEB-targeting drugs. Due to its influence on multiple cellular pathways, non-specific activation may lead to excessive lysosomal activity, disrupt cellular balance, and cause unintended side effects. Persistent TFEB activation could overdrive autophagy, potentially causing damage to healthy proteins and organelles, and this can also impair immune function, increasing the risk of autoimmune diseases 132. Additionally, TFEB-targeting drugs often struggle to cross the blood-brain barrier, potentially limiting their effectiveness in treating brain-related diseases. Therefore, further research is essential to optimize drug delivery systems and formulations for stability and targeted action, particularly for brain-specific therapies.

Interestingly, natural compounds such as berberine 133, rifampicin 134, curcumin 118, genistein 135, and kaempferol 136 have shown potential as TFEB activators, which aligns with our findings on TFEB modulation as a therapeutic approach 137, 138. These compounds may offer alternative or complementary strategies for enhancing TFEB activity with potentially fewer side effects due to their natural origin and inherent bioactivities. However, similar challenges with bioavailability and blood-brain barrier penetration remain.

To address these challenges, it is crucial to develop drugs that finely modulate TFEB activation. Nanotechnology-based approaches for targeted tissue delivery and ligand-based strategies to activate drugs within specific cells or tissues are required 139, 140. Incorporating natural TFEB activators into advanced delivery systems, such as nanoparticles, may further enhance their therapeutic potential by improving stability, targeting efficiency, and brain-specific delivery. Futures studies should investigate the integration of these natural compounds into TFEB-targeting strategies to complement synthetic approaches and overcome existing barriers.

In summary, this review highlights that 1) lysosomal dysfunction caused by genetic causes (*PSEN1/2* mutations, *APOE4* and variants of *BIN1, PICALM* or* SOLR1*), aging, or other factors such as accumulated Aβ or Tau fibril plays a critical role in initiating or amplifying the AD disease process. This dysfunction is associated with faulty acidification, impaired autophagy, and release of lysosomal hydrolases, and 2) therapeutic strategies focused on improving lysosomal stress, lysosomal repair, replacement, reformation, or lysophagy through pharmacologic agents or genetic measure that activate TFEB or related genes may provide new approaches to retard, halt, or reverse disease progression and to treat AD. Future research should focus on translating these findings into safe and effective clinical interventions that provide hope to patients with AD.

## Figures and Tables

**Table 1:** Altered genes or proteins related to lysosomal dysfunction in AD.

Gene name	Functions in autophagy-lysosome	Changes in AD progression	References
mTOR	*mTOR* is a negative regulator of autophagy by phosphorylating and suppressing the ULK1 complex and TFEB.	The expression levels of mTOR/p-mTOR and its downstream targets S6K1/p-S6K1 and RAPTOR/p-RAPTOR were significantly increased in the hippocampus of AD patients' brain in the late stage. In the 3xTg-AD mouse model, increased mTOR activity, indicated by elevated phosphorylated p70S6K levels, is observed in the cortex and hippocampus at 6 and 12 months of age, correlating with Aβ and Tau pathologies.	18 19 20
NRBF2	*NRBF2*, component of the class III PIK3C3 complex, directly interacts with ATG14L, thereby boosting the activity of ATG14L-associated VPS34 kinase, initiating autophagy.	*In the AD human brain,* NRBF2-related kinase complexes, BECN1-PIK3C3 was significantly downregulated specifically in the parahippocampal gyrus. In the 5xFAD AD mouse model, reduced expression levels of NRBF2 may linked to AD pathology, as overexpressing NRBF2 decreases APP-CTFs and Aβ levels.	22 23
CTSD	CTSD is a lysosomal protease belonging to the aspartic proteas and crucial for degrading AD-associated proteins such as APP and Tau.	Mutations in the *CTSD* are associated with an increased risk of AD, in men but not in women. *CTSD* deletion in APP transgenic mice increases intracellular Aβ aggregates and induces severe early onset tauopathy with hyperphosphorylated tau accumulation, independent of human tau overexpression.	25 26
CTSE	CTSE is a lysosomal protease belonging to the aspartic proteas and crucial for degrading AD-associated proteins such as APP and Tau.	Increased protein levels CTSE were observed in the cortex of AD patients and in the hippocampus of 6-month-old APP KI mice, suggesting its potential relevance in the pathology of AD.	27
		CTSE has emerged as a novel biomarker of FAP.	28
PSEN1	Mutations of *PSEN1* disrupt lysosome acidification, leading to markedly impaired autophagy and causing early onset of familial AD.	Defect in lysosomal acidification occurred in *PSEN1* knockdown, KO, and *PSEN1* mutant cells.	29 30
PGRN	Progranulin regulates cathepsin maturation, BMP levels, and lysosomal acidity.	Progranulin mutation is a risk factor for AD progression. Progranulin level in CSF or serum is a diagnostic/prognostic factor in AD.	32 34 35 36
SORL1	SORL1 helps in the sorting and trafficking of APP between the endosome, Golgi complex, and lysosome.	SORL1 protects APP from amyloidogenic processing and guides Aβ to lysosome for degradation.	37
BIN1	BIN1 transports BACE1 to lysosome for degradation to inhibit Aβ generation. BIN1 interacts with Tau.	*BIN1* mutants increase amyloid burden and attenuates GFAP induction in glial cells. BIN1 decreases Tau load in the cortex.	38 40
PICALM	PICALM controls clathrin-coated endocytic vesicle formation, clathrin-mediated endocytosis (CME), and autophagy through endocytosis of VAMP proteins.	A *PICALM* variant is associated with cortical thickness and CSF Aβ42 level. Endothelial PICALM expression is reduced in the brains of AD patients. Endothelial PICALM deficiency impairs clearance and enhances Aβ pathology.	41 42 43

**Table 2:** Autophagy/lysosomal enhancers in AD.

Drugs or Chemicals	Mechanism and effects	References
Atractylenolide III, Ferulic acid and Paeoniflorin	Activation of the AMPK/ULK1/TFEB autophagic signaling pathway.	111 112
	Inhibition of autophagy and neuroinflammation in BV2 microglial cells.	
Caudatin	Activates PPARα.	113
	Improves cognitive dysfunction & enhances lysosomal degradation of Aβ and p-tau aggregates in 3xTg-AD model mice.	
Celastrol	mTORC1 inhibition.	114 115
	Promotes the degradation of p-Tau aggregates in P301S AD model mice.	
Corynoxine	Activating TFEB/TFE3 by inhibiting AKT/mTOR signalling and stimulating lysosomal Ca^2+^ release via TRPML1	116
	Improves learning and memory function in 5xFAD AD mice.	
Crocetin	Activating the STK11/LKB1-mediated AMPK pathway.	117
	Reducing Aβ levels and neuroinflammation, improving memory function in 5xFAD AD mice.	
Curcumin-analog compound C1	Direct binding to and activation of TFEB.	118
	Improves synaptic and cognitive function and reduced Aβ and tau aggregates in P301S, 5xFAD and 3xTg AD mice models.	
Hederagenin	Enhanced expressions of TFEB, LC3II/LC3I, LAMP2A.	119
	Improves cognitive impairment and pathological changes by promoting autophagy in APP/PS1 AD mice.	
Ibudilast	Inhibition of mTORC1 and activation of TFEB.	120
	Improves spatial learning and memory, and increases clearance of Aβ plaque and tau protein aggregates in Aβ/Tau in 344-AD rats.	
Latrepirdine	Stimulates mTOR- and ATG5-dependent autophagy	121
	Reducing Aβ deposition, and prevents further behavioral decline in TgCRND8 mice expressing a double mutant form of APP 695 (KM670/671NL+V717F)	
LH2-051	Promotion of TFEB activation and lysosome biogenesis via inhibition of DAT.	122
	Improves learning, memory, and cognitive function in APP/PS1 AD mice.	
ML246	Activating BECN1.	123
	Aβ removal and memory in 5xFAD AD mice.	
ML-SA1	Activation of TRPML1 endolysosomal Ca2+ signaling	124
	Rescued enlargement and perinuclear clustering of endolysosomes, autophagic vesicle accumulation, and early endosomal enlargement in primary neurons.	
Oleoylethanolamide, KDS-5104	Promotion of TFEB-lysosomal function in a PPARα -dependent but mTORC1-independent manner.	125
	Reduces reactive gliosis and Aβ pathology, and rescues cognitive impairment in 5xFAD AD mice.	
Spermidine	Promotion of eIF5A hypusination	126 127
	Reduction in Aβ deposition and neuroinflammation in APP/PS1 AD mouse model.	
Thonningianin A	Activation of the AMPK/ULK1 and Raf/MEK/ERK pathways	128
	Improving cognitive function, reducing Aβ expression, and decreasing neuronal apoptosis in microglial cells treated with Aβ.	
Trehalose	Activation of TFEB through mTOR-independent, FOXO1-dependent manner	129
	Reduction in Aβ deposition in the hippocampus of APP/PS1 mice	
Uric Acid	Activation of microglia and upregulation of the autophagy-related proteins such as LC3II/I ratio, Beclin-1, and LAMP1	130
	Improves cognitive function in APP/PS1 mouse model of AD.	

## Abbreviations

AD: Alzheimer's disease
ALIX: alg-2-interacting protein x
ALR: autophagic lysosomal reformation
ALS: amyotrophic lateral sclerosis
AMPK: amp-activated protein kinase
APOE4: apolipoprotein e4
APP: amyloid-β precursor protein
ASC: apoptosis-associated speck-like protein containing a card
ATG14L: autophagy-related protein 14-like protein
ATG7: autophagy-related 7
*ATG8*: autophagy-related protein
Aβ: amyloid β
BMP: bis(monoacylglycerol)phosphate
CHMP2A: charged multivesicular body protein 2a
CHMP2B: charged multivesicular body protein 2b
CHMP6: charged multivesicular body protein 6
CLEAR: coordinated lysosomal expression and regulatory
CME: clathrin-mediated endocytosis
CNS: central nervous system
CSF: cerebrospinal fluid
CTF: c-terminal fragment
CTSB: cathepsin b
CTSD: cathepsin d
CTSE: cathepsin e
DAT: dopamine transporters
eIF5A: eukaryotic translation initiation factor 5a-1
ER: endoplasmic reticulum
ESCRT: endosomal sorting complex required for transport
FAP: familial amyloidotic polyneuropathy
FOXO1: forkhead box protein o1
FTD: frontotemporal dementia
GABARAP: gamma-aminobutyric acid receptor-associated protein
Hsp70: heat shock protein 70
HSPG: heparan sulfate proteoglycan
IFN-γ: interferon-γ
IL-1β: interleukin-1β
iPSCs: induced pluripotent stem cells
KI: knockin
KO: knockout
LAMP2: lysosome-associated membrane glycoprotein 2
LPS: lipopolysaccharide
LSR: lysosomal stress response
LRP1: low density lipoprotein receptor-related protein 1
mTOR: mammalian target of rapamycin
NDP52: nuclear dot protein 52
NFTs: neurofibrillary tangles
*NLRP3*: NLR family pyrin domain containing 3
NRBF2: nuclear receptor binding factor 2
OPTN: optineurin
OSTM1: osteoclastogenesis associated transmembrane protein1
PICALM: phosphatidylinositol-binding clathrin assembly protein
PLEKHM1: pleckstrin homology domain containing protein family member 1
*PPARα*: peroxisome proliferator-activated receptor α
PRKAA1: protein kinase amp-activated catalytic subunit alpha 1
PS1: presenilin-1
p-Tau: phosphorylated tau
RAPTOR: regulatory-associated protein of tor
ROS: reactive oxygen species
RIPK1: receptor-interacting protein kinase-1
RyR: resident ryanodine receptor
S6K1: *S6* kinase 1
SAPs: saposins
SNARE: soluble N-ethylmaleimide-sensitive factor attachment protein receptors
SNP: single nucleotide polymorphism
SORL1: sortilin-related receptor 1
RIPK3: receptor-interacting protein kinase-3
STK11/LKB1: serine/threonine kinase 11
TAX1BP1: tax1 binding protein 1
TBC1D15: tbc1 domain family member 15
TFEB: transcription factor eb
TNFR1: tumor necrosis factor receptor-1
TPC2: two-pore channel 2
TRAIL: TNF-related apoptosis-inducing ligand
TRIM16: tripartite motif-containing 16
TRIM22: tripartite motif-containing 22
TRPML1: transient receptor potential mucolipin 1
ULK1: unc-51-like autophagy-activating kinase
VAMPS: vesicle-associated membrane proteins
v-ATPase: vacuolar ATPase
